# Factor Analyses and Validity of the Transplant Evaluation Rating Scale (TERS) in a Large Sample of Lung Transplant Candidates

**DOI:** 10.3389/fpsyt.2020.00373

**Published:** 2020-04-30

**Authors:** Mariel Nöhre, Georgios Paslakis, Özgür Albayrak, Maximilian Bauer-Hohmann, Jan Brederecke, Daniela Eser-Valeri, Igor Tudorache, Martina de Zwaan

**Affiliations:** ^1^Department of Psychosomatic Medicine and Psychotherapy, Hannover Medical School, Hannover, Germany; ^2^Biomedical Research in Endstage and Obstructive Lung Disease Hannover (BREATH), German Center for Lung Research (DZL), Hannover Medical School, Hannover, Germany; ^3^University Health Network, Toronto General Hospital, Toronto, ON, Canada; ^4^Department of Psychiatry, University of Toronto, Toronto, ON, Canada; ^5^Department of Pediatric Cardiology and Intensive Care, Hannover Medical School, Hannover, Germany; ^6^Department of Psychiatry, Ludwig Maximilians University, Munich, Germany; ^7^Department of Cardiac, Thoracic, Transplant, and Vascular Surgery, Hannover Medical School, Hannover, Germany

**Keywords:** lung transplantation, Transplant Evaluation Rating Scale (TERS), psychosocial evaluation, confirmatory factor analysis, mental disorders

## Abstract

**Objective:**

It is well known that the occurrence of mental disorders is more common in lung transplant candidates compared to the general population. After transplantation mental disorders may negatively affect quality of life, adherence to immunosuppressive medication, as well as overall survival. Therefore, the identification of patients at risk is of utmost importance and in Germany pre-transplant psychosocial evaluation of the patients is required. To ensure high quality and comparability of these assessments, the use of psychometrically sound instruments is recommended. We applied the Transplant Evaluation Rating Scale (TERS), a broadly used expert interview. Two research groups have detected a two-factor structure of the TERS in different transplant samples; however, with slightly different results. The present study investigated which of the models would fit best in our sample of lung transplant patients. Additionally, we assessed convergent and predictive validity of the best fitting model to evaluate its clinical usefulness.

**Methods:**

Between 2016 and 2019, 390 lung transplant candidates were evaluated and included in the study. The median age was 53 years and 54% were male. TERS interviews were conducted by trained medical doctors and psychologists. The participants completed questionnaires assessing quality of life and levels of depression and anxiety. Transplant- and disease-specific variables (lung disease, listing date, oxygen use) were taken from the patient charts. Confirmatory factor analysis was used to test the two proposed TERS-models in the present sample.

**Results:**

The two-factor structure of the TERS reported by Hoodin and Kalbfleisch fit our sample best, showing good psychometric properties. The factor “emotional sensitivity” was highly correlated with quality of life and measures of psychosocial health while the factor “defiance” correlated with obstructive lung disease and older age but not with quality of life. The two factors showed differential predictive validity with regard to time until listing and pulmonary-specific quality of life 1 year after transplantation.

**Conclusions:**

The two factors showed good psychometric properties, and differential convergent and predictive validity. However, further studies concentrating on the predictive value of the TERS and its factors regarding somatic outcomes (mortality, graft functioning) are required.

## Introduction

Lung transplantation is the final treatment option for patients with end-stage lung disease. It is well known that lung transplantation is an exhausting as well as a physically and mentally challenging procedure for the patients. Mental disorders are more common in lung transplantation candidates than in the general population (1). After transplantation, mental health and quality of life generally improve (2). However, as mental disorders after transplantation may deteriorate quality of life, adherence to immunosuppressive medication, as well as overall survival (3–5), detecting patients at risk is essential. Mental disorders are no contradiction regarding listing for transplantation. However, patients suffering from mental disorders before transplantation may require psychosocial support and should be treated and followed appropriately. Based on that, the guideline of the German Medical Association concerning lung transplantation dictates that lung transplant candidates should be evaluated by a mental health professional before transplantation (6).

To ensure a high quality and comparability of the psychosocial evaluation procedure, the use of validated instruments is supported. One broadband instrument is the Stanford Integrated Psychosocial Assessment for Transplantation (SIPAT) (7). The SIPAT has been recently developed and first studies show good predictive validity regarding psychosocial and clinical outcomes after organ transplantation (7, 8). However, so far there are no findings regarding its predictive validity in patients after lung transplantation available. Additionally, there is to date no validated German version of the instrument available. Therefore, in our study, we used the Transplant Evaluation Rating Scale [TERS, (9)], a well-established expert interview for the assessment of psychosocial functioning before organ transplantation. It covers 10 distinct domains of psychosocial functioning considered relevant for adjustment to transplantation and its consequences. Initially, the TERS was designed as a unidimensional instrument. However, Hoodin and Kalbfleisch (10) detected two factors, which they called “emotional sensitivity” and “defiance” in a sample of 345 bone marrow transplant recipients using exploratory factor analysis (EFA). In 2018, Zimmermann et al. (11) also applied EFA in 75 lung transplant candidates. They were able to detect two factors as well, and also referred to them as “emotional sensitivity” and “defiance” even though their subscales did not entirely match the original subscales. In the model suggested by Hoodin and Kalbfleisch (10) the domains “personality disorders,” “substance use/abuse,” “compliance,” “health behaviors,” “quality of family and social support,” and “history of coping” loaded on the factor “defiance” while the other ones (“past or current mental disorders,” “current coping with disease and treatment,” “quality of affect and, mental/cognitive status [past and present]”) loaded on the factor “emotional sensitivity.” In Zimmermann et al.’s (11) model the domains “mental status” and “personality disorder” loaded on the other factor.

On that basis, the aim of the present study was to evaluate which of the proposed two-factor models is the most suitable for our sample of lung transplant candidates. Additionally, we explored the convergent validity of the two factors regarding measures of health-related quality of life (HRQoL), measures of depression and anxiety, and measures of lung disease severity. Also, TERS scores were compared between disease groups as well as risk groups as described by Hoodin and Kalbfleisch (10) and Yost et al. (12). Finally, we examined the predictive value of the TERS factors with regard to listing for lung transplantation and with regard to pulmonary quality of life 1 year after transplant. So far, information regarding the predictive validity of evaluation instruments for organ transplant candidates is scarce and further research in this field is much needed (13).

## Methods

### Participants

Between January 2016 and April 2019, 390 lung transplantation candidates presented for psychosocial evaluation prior to enlistment at Hannover Medical School and were included in the study. Levels of psychosocial functioning were routinely assessed using the TERS. The TERS interview was conducted according to a structured protocol by residents in psychosomatic medicine and psychologists in training for psychotherapy. We developed an interview guideline to standardize the evaluation interview and to make sure that the important information is collected. The examiners were experienced in the treatment of patients before and after organ transplantation. When insecurities regarding the correct rating of a patient occurred, the case was discussed in a team meeting. Additionally, patients were asked to fill out several questionnaires. The participants had to be at least 18 years of age and possess sufficient German language skills. The study was approved by the Institutional Ethics Board of Hannover Medical School (no 3120-2016), and all patients gave written informed consent.

Two nonrandom subsamples in our original sample of 390 patients were used to assess predictive validity: The first subsample consists of 262 patients who had been listed until April 2019. At this time point 195 patients were already transplanted. During the first year after transplantation 9 patients (4.6%) had died. Follow-up examinations were available for 109 patients (55.9%) who completed the Pulmonary-Specific Quality-of-Life Scale (PQLS) 1 year after transplantation. Follow-up examinations of 77 patients (39.5%) were missing. While some of the patients did not participate in the follow-up examination, others have not yet completed the first year after transplantation. Both subsamples (262 patients and 109 patients) did not differ significantly from the overall sample in sex, age, lung disease, and TERS scores.

### Transplant Evaluation Rating Scale

The Transplant Evaluation Rating Scale (9) is an expert interview for the assessment of psychosocial functioning prior to organ transplantation. The TERS is comprised of 10 distinct domains of psychosocial functioning considered relevant for adjustment to transplantation and its consequences: 1) current or past mental disorders (axis 1 according to DSM-IV), 2) personality disorder (axis 2 according to DSM-IV), 3) substance use/abuse, 4) compliance, 5) health behaviors, 6) quality of family and social support, 7) history of coping, 8) current coping with disease and treatment, 9) quality of affect and, 10) mental/cognitive status (past and present). Each of the ten items is rated by a clinician on a three-point scale (1 = minimal/mild, 2 = moderate, 3 = severe impairment). To reflect the importance of the respective domain for the overall level of psychosocial functioning, each item rating is multiplied by an *a priori* assigned weight (ranging from 1 to 4) and the items are added up to calculate the total (weighted) score ranging from 26.5 to 79.5. Higher scores represent greater impairment in the levels of psychosocial functioning. In addition, patients were stratified as suggested by previous studies (10, 12) according to their score into low-, moderate-, and high-risk groups with scores 26.5–29, 29.5–37, and 37.5–79.5, respectively.

### Generic Health-Related Quality of Life

We used the Short-Form 8 Health Survey (SF-8), a short version of the SF-36 Health Survey, to measure generic HRQoL (14–16). It consists of two subscales, the Physical Component Scale (PCS) and the Mental Component Scale (MCS). Both are standardized combined scores with a mean of 50 and a standard deviation of 10 in the US general population. Cronbach’s α for the total SF-8 was 0.778.

### Symptoms of Depression and Anxiety

We used the nine-question depression scale from the Patient Health Questionnaire (PHQ-9) to measure symptoms of depression (17, 18). Each of the nine items is scored on a four-point Likert-scale ranging from 0 (“not at all”) to 3 (“nearly every day”) leading to a total score between 0 and 27. Higher values are indicative of a higher level of depressive symptoms. Cronbach’s α in the present sample was 0.769.

The German version of the Generalized Anxiety Scale (GAD-7) was used to measure levels of anxiety (19, 20). The scale consists of seven items which are scored on a four-point Likert scale between 0 (“not at all”) to 3 (“nearly every day”). This leads to a total score between 0 and 21. Higher values in the total scores correspond with higher levels of anxiety symptoms. Cronbach’s α in the present sample was 0.830.

### Demographics and Measures of Disease Severity

Patients were asked to report their age and sex. Four groups were defined based on the patient’s lung disease: obstructive lung disease (e.g. chronic obstructive lung disease, emphysema, bronchiectasis), restrictive lung disease (e.g. pulmonary fibrosis), cystic fibrosis, and other lung diseases (e.g. pulmonary vascular disease, idiopathic pulmonary arterial hypertension, sarcoidosis). Functional capacity was measured with supplemental oxygen use at rest (L/min). A higher amount of supplemental oxygen was considered as being indicative of a higher degree of severity of the lung disease (21).

### Listing Status

Listing status was evaluated for all participants in April 2019 (n = 262 were listed) and we calculated the time between presenting for psychosocial evaluation and listing date for each patient. The information was taken from the electronic patient charts.

### Pulmonary-Specific Quality-of-Life Scale 1 Year After Transplant

The Pulmonary-Specific Quality-of-Life Scale (PQLS) is a self-report questionnaire assessing HRQoL specifically in patients prior to and after lung transplantation (22–24). Each of the 25 items is rated on a five-point-Likert-scale ranging from 1 (“not at all”) to 5 (“most of the time”). A total score between 25 and 125 can be reached. Higher values are indicative of lower HRQoL. The scale comprises three subscales (“task interference,” “psychological,” and “physical”), which focus on different aspects of HRQoL. For this study, the German version was used (24). Cronbach’s α was 0.805 for the subscale “task interference,” 0.829 for the subscale “psychological,” 0.881 for the subscale “physical,” and 0.866 for the total score.

### Statistical Analyses

Confirmatory factor analysis (CFA) was used to evaluate both models. CFA models were estimated using WLSMV (weighted least squares with mean and variance adjusted) estimation that utilizes diagonally weighted least squares (DWLS) as well as mean and variance adjusted test statistics. WLSMV was chosen as it is recommended for the use with ordinal and skewed data (25) and multiple items in the present sample showed high levels (> 2) of skewness. All CFA related analyses were performed using the lavaan package (26) for R (27). General model fit was assessed using multiple criteria: Comparative fit index (CFI) for fit relative to a null model complemented with the standardized root mean square residual (SRMR) and the root mean square error of approximation (RMSEA) for overall fit. According to Hu and Bentler (28), the criteria for good model fit are CFI >0.95 (0.90 is acceptable), SRMR <0.08, and RMSEA <0.06 (0.09 is acceptable). As the χ2-test statistic is very sample size sensitive (29), it is not considered in the evaluation process and only displayed for reasons of completeness. The two models are not nested, making a direct model comparison generally difficult. As the Bayesian Information criterion [BIC; (30)] and Vuong’s test (31) are not available with WLSMV estimation in R, individual model fit was evaluated and the two models were then compared only descriptively, taking additional factors like inter-factor correlation into account.

Spearman correlations were used to explore the associations between the TERS total score as well as the two TERS factor scores and measures of HRQoL, levels of anxiety and depression, and time until listing. Correlation coefficients ≥0.1 were interpreted as a low correlation, coefficients ≥0.3 as a moderate correlation, and coefficients ≥0.5 as a strong correlation. Thanks to great efforts in the process of data collection, there was no missing data in the sample. Thus, no participants had to be excluded and no measures had to be taken to account for missing data. As the data were not normally distributed, we conducted Kruskal-Wallis-tests to compare TERS total and factor scores between disease groups and TERS risk groups. Dunn-Bonferroni *post-hoc* tests were used to evaluate differences between disease and risk groups.

Finally, multiple linear regression analyses were conducted with the time since listing, the PQLS total score and the three PQLS subscale scores 1 year after transplantation as dependent variables and age, sex, lung disease, and the two TERS factors as independent variables.

For the questionnaires a maximum of 10% missing data was allowed per questionnaires. This corresponds to one missing item in most of the applied questionnaires. The missing item was replaced with the mean value.

Statistical analyses were performed using IBM^®^ Statistical Software Package of Social Science Statistics (SPSS^®^, Chicago, IL, USA) version 26 and R 3.4.4, as appropriate. We considered p < 0.05 statistically significant.

## Results

### Description of the Sample

Participants’ characteristics can be found in **Table 1**. Three hundred ninety patients participated in the study. The sample consisted of 179 women (45.9%) and 211 men (54.1%). The median age was 53 years [Interquartile range (IQR) 15]. Twenty-eight point five percent of the participants suffered from restrictive lung disease, the most common lung disease in our sample, followed by obstructive lung disease (27.6%), cystic fibrosis (26.8%), and others (17.4%).

**Table 1:** Characteristics of study participants.

Parameter	
N (%)	390 (100%)
Age in years	
mean (SD)	49.6 (12.2)
median (IQR)	53.0 (15)
Female gender, n (%)	179 (45.9%)
Lung disease, n (%)	
Obstructive	107 (27.6%)
Cystic fibrosis	104 (26.8%)
Restrictive	111 (28.5%)
Other	68 (17.4%)
Listed for transplantation (April 2019)	262 (67.2%)
One-year follow-up available	109 (27.9%)
Oxygen use at rest (L/min)	
mean (SD)	2.4 (1.8)
median (IQR)	2.0 (1.5)
GAD-7	
mean (SD)	3.8 (3.3)
median (IQR)	3.0 (5.0)
PHQ-9	
mean (SD)	6.8 (4.2)
median (IQR)	6.0 (7.0)
SF-8, PCS	
mean (SD)	32.7 (7.6)
median (IQR)	32.3 (9.6)
SF-8, MCS	
mean (SD)	46.2 (10.9)
median (IQR)	47.0 (16.5)

### Factor Structure

As shown in **Table 2**, both models showed acceptable to good model fit regarding CFI and RMSEA. Nonetheless, both models resulted in an SRMR above the recommended threshold. As the SRMR represents the standardized difference of the observed correlations from the predicted correlations, the residual covariances were consulted. This revealed that both models were not sufficiently taking care of a number of correlations (Residual-covariance > |.1|).

**Table 2:** Model fit indices of the CFA-models.

	χ^2^	*p* (χ^2^)	df	CFI	SRMR	RMSEA (CI)
Hoodin and Kalbfleisch (8)	58.15	.006	34	.94	.12	.04 (.02–.06)
Zimmermann et al. (9)	64.08	.001	34	.93	.12	.05 (.03–.07)
Hoodin and Kalbfleisch (8) + 7 freed error covariances	26.14	.511	27	1	.08	.00 (.00–.38)
Zimmermann et al. (9) + 6 freed error covariances	32.02	.274	28	.99	.09	.02 (.00–.05)

CFI, comparative fit index; SRMR, standardized root mean square residual; RMSEA, root mean square error of approximation; CI, confidence interval.

In order to see if model fit could be improved, modification indices were considered following Bentler and Chou’s (32) remark that completely uncorrelated error terms are seldom appropriate regarding real data. The procedure was as follows and was carried out for both models separately: The highest error covariance was included and then a likelihood-ratio test determined if this improved the model fit in a statistically significant way. The process was then stopped when freeing another parameter did not improve the model fit in a statistically significant way.

This resulted in freeing seven parameters in the model by Hoodin and Kalbfleisch which drastically improved the model fit as is shown in **Table 2** (see also **Supplementary Table 1**). Seven parameters were freed in the model by Zimmermann et al. and this did improve model fit nearly equally well. From this point of view, both models represent the data in a very accurate way with the SRMR in Zimmermann et al.’s model still above the threshold nonetheless.

**Figure 1:**
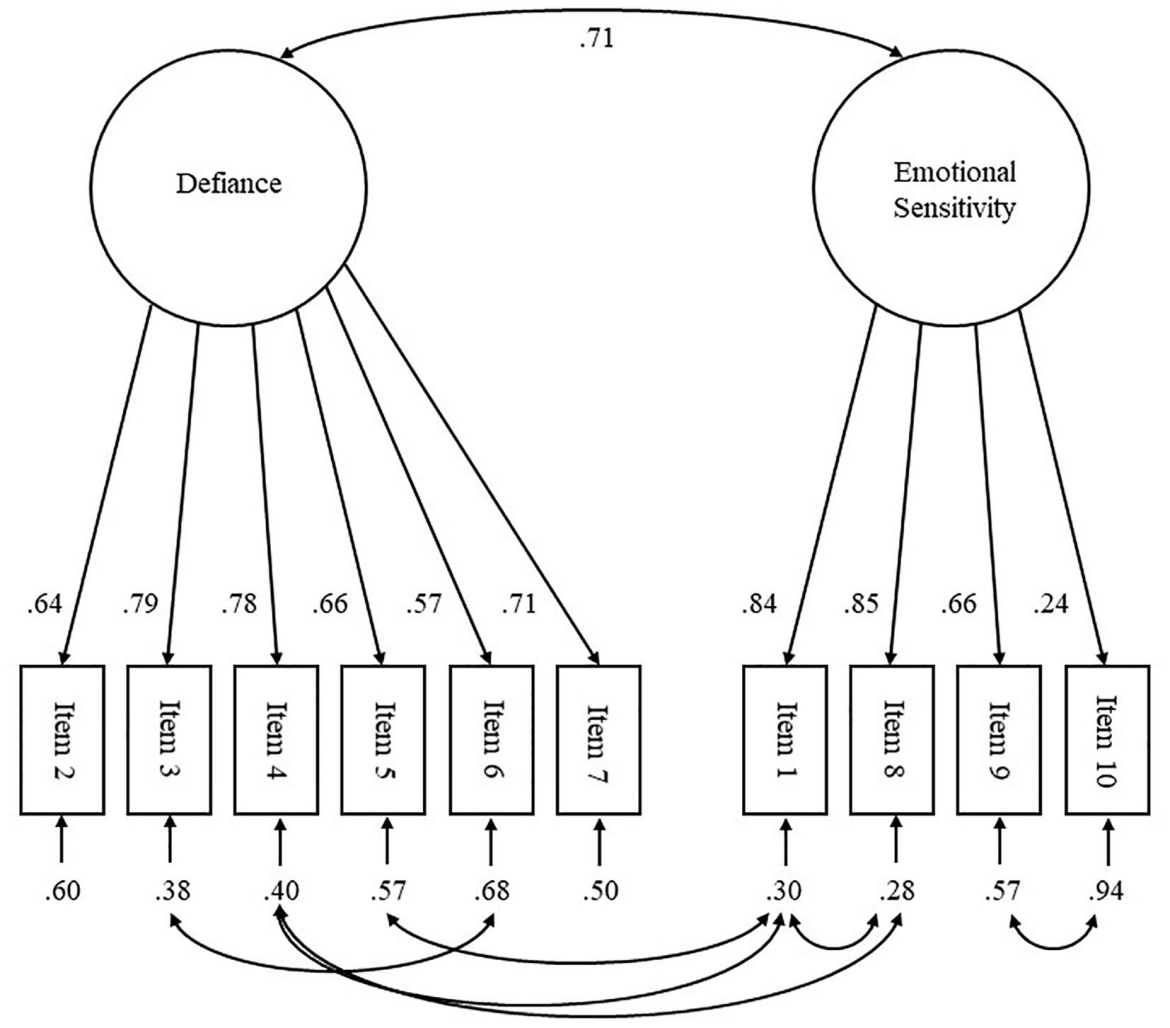
Standardized factor loadings of Hoodin and Kalbfleisch’s (10) TERS two-factor model with 7 freed error covariances. Squares represent TERS items, circles indicate the two associated latent factors. All loadings are statistically significant (p < .05).

Both models showed a high factor inter-correlation (Hoodin et al.’s Model r =.71 and Zimmermann et al.’s Model r =.94). This suggested that the constructs “defiance” and “emotional sensitivity” are considerably overlapping in both models but leave Hoodin and Kalbfleisch’s (10) model still below the threshold (r =.85) proposed by Cohen et al. (33). An item allocation according to Hoodin and Kalbfleisch (10) thus results in sufficiently differentiated factors and a slightly better fit than Zimmermann et al.’s model (11) when fit indices are considered descriptively. It was thus decided that Hoodin and Kalbfleisch’s model with the added error covariances (see **Figure 1**) was representing the data in the present sample best. Further analyses were performed with the TERS factors as defined by Hoodin and Kalbfleisch (10).

Lastly, item 10 (“mental status”) showed a considerably low standardized factor loading of .28 (see **Figure 1**) that is far below the threshold for useful items of .45 proposed by Comrey and Lee (34). However, it was decided to keep it, in order to preserve the practical utility item 10 ultimately contributes to the TERS from a practical point of view.

### Validity of the TERS Factors “Defiance” and “Emotional Sensitivity”

#### Convergent Validity of the TERS Factors With Measures of Quality of Life, Depression, and Anxiety

In **Table 3**, the relationships between the TERS total score and the two factors with levels of HRQoL, and levels of depression and anxiety are reported. The TERS factor “emotional sensitivity” correlated significantly and negatively with the MCS of the SF-8 (r = −0.318, p < 0.01). Additionally, there was a significant and positive correlation with symptoms of depression (r = 0.306, p < 0.01) and anxiety (r = 0.321, p < 0.01). No statistically significant correlation could be found with the PCS of the SF-8.

**Table 3:** Relationship between the TERS and its two factors and measures of health-related quality of life, levels of depression and anxiety, sociodemographic parameters and measures of disease severity.

	TERS sum score	“Defiance”	“Emotional Sensitivity”
SF-8			
PCS	−.050	.011	−.095
MCS	−.288**	−.085	−.318**
PHQ-9 (Depression)	.245**	.073	.306**
GAD-7 (Anxiety)	.281**	.119*	.321**
Age	.042	.134**	−.035
Oxygen use at rest (L/min)	.037	−.003	.031
Time until listing	.122*	.148*	.055

SF-8, Short Form 8 Health Survey; PCS, Physical Component Scale; MCS, Mental Component Scale; GAD-7, Generalized Anxiety Scale; PHQ-9, Patient Health Questionnaire-Depression Scale.

*p < .05, **p < .01. Statistically significant results (p < .05 and p < .01) are shown in boldface.

The factor “defiance” correlated significantly and positively only with the level of anxiety (r = 0.119, p < 0.05).

#### Associations With Disease Groups and TERS Risk Groups

For all patients the mean TERS total score was 31.1 (SD 5.2), 11.3 (SD 2.7) for the factor “emotional sensitivity,” and 20.0 (SD 3.4) for the factor “defiance.” When comparing the results between risk groups, the TERS total score as well as the factor scores differed significantly between risk groups (**Table 4**).

**Table 4:** Comparison of TERS total score and subscales between risk groups.

	TERS risk groups [Hoodin and Kalbfleisch (10)]			Statistics
	Low risk N = 179	Moderate risk N = 170	High risk N = 41	Kruskal-Wallis-Test
TERS total score, Mean (SD) [range 26.5–79.5]	27.5 (1.1)^a^	32.5 (2.0)^b^	43.0 (5.0)^c^	X^2^ = 325.05 (df = 2) p < .001
Factor “emotional sensitivity,” Mean (SD) [range 9–27]	9.2 (0.7)^a^	12.6 (1.9)^b^	15.4 (2.8)^c^	X^2^ = 253.62 (df = 2) p < .001
Factor “defiance,” Mean (SD) [range 17.5–52.5]	18.3 (1.2)^a^	20.0 (2.2)^b^	27.5 (4.1)^c^	X^2^ = 154.46 (df = 2) p < .001

Different superscripts (a-c) indicate significant differences in post-hoc test (Dunn-Bonferroni-test).

Additionally, we compared TERS results between lung disease groups (**Table 5**). Patients suffering from obstructive lung disease had significantly higher scores on the TERS total score and the factor “defiance” compared to the other three disease groups.

**Table 5:** Comparison of TERS total score and subscales between lung disease groups.

	Lung disease groups				Statistics
	Obstructive N = 107	CF N = 104	Restrictive N = 111	Other N = 68	Kruskal-Wallis-Test
TERS total score, Mean (SD) [range 26.5–79.5]	**32.8 (5.0)^a^**	31.1 (5.1)^b^	30.8 (5.5)^b^	30.2 (4.4)^b^	X^2^ = 22.19 (df = 3) p < .001
Factor “emotional Sensitivity,” Mean (SD) [range 9–27]	11.7 (2.6)	11.5 (2.7)	11.1 (2.8)	10.9 (2.5)	X^2^ = 4.27 (df = 3) p =.234
Factor “defiance,” Mean (SD) [range 17.5–52.5]	**21.2 (3.6)^a^**	19.6 (3.3)^b^	19.6 (3.5)^b^	19.2 (2.8)^b^	X^2^ = 34.78 (df = 3) p < .001

Different superscripts (a-b) indicate significant differences in post-hoc test (Dunn-Bonferroni-test).

CF, cystic fibrosis. Statistically significant results (p < .05 and p < .01) are shown in boldface.

#### Association With Age, Sex, and Oxygen Use at Rest

Higher age was significantly but weakly correlated with a higher score in the factor “defiance” (r = 0.134, p < 0.01). There was no significant correlation between age and the TERS total score and the factor “emotional sensitivity.” There were no statistically significant differences in the TERS total score and the two factors between female and male participants. Neither the TERS total score nor the two factors were significantly associated with supplemental oxygen use at rest as a measure for the disease severity.

#### Predictive Validity

Time until listing was investigated in a subset of 262 patients who were listed until April 2019. Time until listing was significantly longer in patients with higher values in the TERS total score (r = 0.122, p < 0.05) and higher values in the factor “defiance” (r = 0.148, p < 0.05). Linear regression analysis revealed that this was not merely explained by age, sex, and lung disease (**Supplementary Table 2**).

We investigated if the TERS factors would differentially predict pulmonary-specific quality of life 1-year post-transplant for a subset of 109 patients in our original sample of 390 patients. The two factors exhibited differential predictive validity to quality of life at 1-year post-transplant. After adjusting for age, sex, and lung disease, the factor “emotional sensitivity” predicted task interference (the model explaining almost 24% of the variance) while the factor “defiance” predicted physical functioning (the model explaining 7% of the variance). None of the factors predicted psychological functioning at 1-year follow-up (**Table 6**).

Table 6Regression analyses with PQLS total and subscale scores as dependent variables.

**Table:** 

***a) PQLS total score***							
	Non standardized coefficient		Standardized coefficient			95% confidence interval	
	B	SE	β	T	Sig.	Low	High
**(Constant)**	−6.192	11.778		−.526	.600	−29.552	17.168
**Age**	.348	.104	.307	3.344	**.001**	.142	.554
**Sex**	−.111	2.819	−.004	−.039	.969	−5.702	5.480
**Lung disease**	2.713	1.296	.186	2.093	**.039**	.143	5.283
**Emotional sensitivity**	1.405	.562	.232	2.501	**.014**	.291	2.519
**Defiance**	.602	.423	.133	1.425	.157	−.236	1.440

F = 5.51 (df = 103), p < .001; adjusted R^2^ =.17; SE, standard error

**Table:** 

*b) PQLS Task Interference*							
	Non standardized coefficient		Standardized coefficient			95% confidence interval	
	B	SE	β	T	Sig.	Low	High
**(Constant)**	−12.071	5.621		−2.147	.034	−23.227	−.916
**Age**	.183	.049	.337	3.709	**.000**	.085	.281
**Sex**	.588	1.340	.040	.439	.661	−2.070	3.247
**Lung disease**	1.830	.608	.262	3.008	**.003**	.623	3.038
**Emotional Sensitivity**	.766	.262	.262	2.919	**.004**	.245	1.287
**Defiance**	.305	.200	.139	1.525	.130	−.092	.702

F = 7.41 (df = 98), p < .001; adjusted R^2^ =.24; SE, standard error

**Table:** 

*c) PQLS Psychological Functioning*							
	Non standardized coefficient		Standardized coefficient			95% confidence interval	
	B	SE	β	T	Sig.	Low	High
**(Constant)**	8,943	4,122		2,169	.032	.767	17.118
**Age**	−.005	.036	−.015	−.149	.882	−.078	.067
**Sex**	−.143	.987	−.015	−.144	.885	−2.099	1.814
**Lung disease**	.186	.454	.041	.410	.683	−.714	1.085
**Emotional sensitivity**	.255	.197	.134	1.297	.197	−.135	.645
**Defiance**	−.064	.148	−.045	−.431	.667	−.357	.230

F = 0.37 (df = 103), p =.87; SE, standard error

**Table:** 

*d) PQLS Physical Functioning*							
	Non standardized coefficient		Standardized coefficient			95% confidence interval	
	B	SE	β	T	Sig.	Low	High
**(Constant)**	−3.783	3.788		−.999	.320	−11.296	3.730
**Age**	.075	.033	.219	2.255	**.026**	.009	.142
**Sex**	.204	.907	.022	.225	.822	−1.594	2.003
**Lung disease**	.260	.417	.059	.623	.535	−.567	1.086
**Emotional sensitivity**	.068	.181	.037	.374	.709	−.291	.426
**Defiance**	.285	.136	.208	2.097	**.038**	.016	.555

F = 2.56 (df = 103), p =.03; adjusted R^2^ =.067; SE, standard error

## Discussion

The main finding of our study was that both two-factor models of the TERS that have been previously described in the literature could be replicated in our sample of lung transplantation candidates, with Hoodin and Kalbfleisch’s (10) model representing the data of our sample best. We came to this conclusion based on different findings: When comparing the fit indices descriptively the Hoodin and Kalbfleisch (10) model showed a slightly better fit. Another aspect supporting the use of the model of Hoodin and Kalbfleisch (10) is its lower factor inter-correlation compared to Zimmermann et al.’s (11) model. This element seems to be important from a clinical perspective. More detailed information is expected to be deduced from the TERS when regarding the two factors compared to the TERS total score. However, this presupposes that both factors do not overlap too much which can only be said for the model of Hoodin and Kalbfleisch (10), supporting its use in our sample (33).

However, one difficult aspect is the low factor loading of domain 10 “mental status” of merely.28 (34). The decision to keep it and not to exclude it from our model was made based on its clinical importance. The domain “mental status” contains information regarding the cognitive impairment of the transplant candidate. As it is known that reduced cognitive functioning after transplantation can interfere with adherence behavior and was described as an independent risk factor for increased mortality in these patients, it should already be taken into account before transplantation (35–37).

Above the fit of the two-factor models we wanted to explore if there was further benefit from a clinical viewpoint concerning the use of the two factors. The factor “emotional sensitivity” showed statistically significant and moderate correlations with measures of anxiety, depression, and psychological aspects of quality of life. These findings are in line with the results reported by Hoodin and Kalbfleisch (10), suggesting that this factor is indicative of the current psychological situation. Overall, a certain amount of affective distress and anxiety in patients with end-stage lung disease may constitute an appropriate response to a serious and life-threatening medical condition. However, excessively elevated pre-transplant TERS “emotional sensitivity” scores might need appropriate interventions to facilitate immediate adjustment.

The factor “defiance” correlated significantly positive but weakly with symptoms of anxiety. Above that, we found a significant positive association with age. This suggests that the factor “defiance” in contrast to “emotional sensitivity” is only to a small degree affected by the current psychological situation. When comparing the TERS scores between lung disease groups, patients suffering from obstructive lung disease showed significantly higher TERS total scores and specifically “defiance” scores than patients in the other disease groups. The “defiance” scale depicts behavioral, social, and cognitive self-regulatory capacities and is most likely indicative for behavioral but less for psychological difficulties. Obstructive lung disease can be a consequence of smoking and other unhealthy behaviors and often occurs in higher age which might be an explanation for the correlation with the factor “defiance.”

There was no correlation between either one of the TERS factors and the TERS total score and oxygen use at rest as an indicator for disease severity and the physical aspects of quality of life as measured with the PCS of the SF-8. This result underlines again, that prior to listing the TERS focuses predominantly on psychosocial aspects independent from the physical constitution (9).

Comparing the TERS risk groups as defined by Hoodin and Kalbfleisch (10) both factor scores increased significantly with increasing TERS scores. These results show that both factors contribute equally to the TERS severity/risk groups.

Based on their longitudinal results, Hoodin and Kalbfleisch (10) reported in patients after bone marrow transplantation (BMT) that higher values in the factor “defiance” seem to be indicative for behavioral difficulties, suggesting that more difficulties after transplantation are expected to appear in patients with a history of drug abuse or in patients already having shown non-adherent behavior in the past. Based on these assumptions, we can hypothesize that the longer time until listing for transplantation in candidates with higher “defiance” scores might be explained by a more intense evaluation requiring more time than the normal evaluation procedure. It might also be due to hesitation of the transplant team to list the patient as a history of drug abuse or non-adherent behavior in the past might undermine adherence with medical treatment. However, there is no detailed information available explaining the duration of the listing process in detail and there is so far no information available on this aspect from other studies in organ transplant recipients, it becomes obvious that there is still need for research in this area.

Finally, the clinical utility in measuring the factors separately is supported by their differential prediction of different aspects of quality of life 1 year after transplant. The “defiance” score was a significant predictor of physical functioning 1 year post-transplant which might be the consequence of persistent long-term behavioral difficulties such as reduced self-care and low adherence after transplantation. This is comparable with the prediction of the Sickness Impact Profile score, a measure of functional impairment 1 year after BMT by the “defiance” score in the study by Hoodin and Kalbfleich (10). The “emotional sensitivity” score was a significant predictor of task interference at the 1-year post-transplant assessment independent of age and lung disease. Since the PQLS is a measure of subjectively perceived health status and quality of life, poor emotional and psychological adjustment to the transplant process might explain the prediction of a subscale which focuses on occupational and social functioning. However, while the “emotional sensitivity” scale was clearly associated with other measures of psychological distress at pre-transplant, it did not predict psychological aspects of quality of life post-transplant.

The strengths of our study are the large consecutive sample of lung transplant candidates and the use of CFAs to test the factor structure of the TERS enabling us to identify the model suggested by Hoodin and Kalbfleisch (10) as the most for our sample. Data collection was prospective and longitudinal. Above that we were able to generate further evidence supporting the construct and predictive validity of the factors of the TERS. As recommended by others there is a need for additional psychometric work in different transplant populations regarding instruments currently being used in practice (13).

However, there are some limitations worth noting. We performed two CFAs to evaluate which model fits best. As to our knowledge there are no statistically sound methods available to test it statistically, we had to decide for one model over the other only descriptively based on the model fit data available. We are well aware that there are other measurements available specifically designed to evaluate physical functioning in patients with chronic lung disease: For example the BODE index, which includes the body mass index (BMI), forced expiratory volume in one second (FEV1), subjective dyspnea, and the six-minutes-walk test (6-MWT) (38). Even the 6-MWT by itself provides is a valid measure of disease status (39). Research suggests that measuring frailty might give insight into the physical constitution of patients suffering from lung diseases (40). We chose supplemental oxygen use at rest since this is a parameter that most patients are well aware of and it is one objective marker for disease severity used also in other studies (41, 42). Additionally, patients included in this study were recruited during psychosocial evaluation before listing for transplantation. Therefore, they represent a special group of patients having already undergone several stages of the enlistment process. It might be possible that our findings cannot be applied unreservedly to patients suffering from end-stage lung disease at earlier treatment stages.

In conclusion, we were able to confirm the two-factor structure of the TERS reported by Hoodin and Kalbfleisch (10) in our sample. Above that, our results show the benefit of measuring the two factors individually, providing more detailed information on the candidate’s psychological and behavioral constitution. While we found a correlation between the subscale “defiance” and longer time until listing for transplantation, the mechanisms explaining this result are not completely understood. The differential prediction of different aspects of quality of life 1 year after transplantation further supports the clinical usefulness of the factor structure. Therefore, further studies concentrating on the predictive value of the TERS taking more detailed medical information into account are required. Future studies should investigate how the TERS and its factors perform as predictors of somatic morbidity and mortality after transplantation.

## Data Availability Statement

The datasets generated for this study are available on request to the corresponding author.

## Ethics Statement

The studies involving human participants were reviewed and approved by Institutional Ethics Board of Hannover Medical School. The patients/participants provided their written informed consent to participate in this study.

## Author Contributions

MZ designed the study. ÖA and MB-H were mainly responsible for data acquisition. MZ, JB, and MN analysed the data, and MN wrote the first draft. All authors contributed significantly to the interpretation of the data and the final version of the manuscript. All authors gave final approval of the version to be published.

## Funding

The study was supported by a grant from Biomedical Research in Endstage and Obstructive Lung Disease Hannover (BREATH) within the German Center for Lung Research (82DZL002A1).

## Conflict of Interest

The authors declare that the research was conducted in the absence of any commercial or financial relationships that could be construed as a potential conflict of interest.

The handling editor declared a past co-authorship with one of the authors, MZ.
