# Supplementary material for: Factor Analyses and Validity of the Transplant Evaluation Rating Scale (TERS) in a Large Sample of Lung Transplant Candidates
Source: Front Psychiatry. 2020 Apr 30;11:373. doi: 10.3389/fpsyt.2020.00373 (PMC7205021; doi:10.3389/fpsyt.2020.00373)
Supplement: Supplementary file 1 [file Table_1.pdf]

1 Supplementary Material

Supplementary Table 1: Factor loadings for the 2-factors according to Zimmermann et al. (11)

Item	Factor “emotional sensitivity”	Item	Factor “defiance”
1	.578	3	.742
2	.653	4	.788
8	.728	5	.611
9	.707	7	.707
		10	.236
		6	.556

Supplementary Table 2: Regression analyses with time since listing as dependent variable

	**Non standardized coefficient**		**Standardized coefficient**	**T**	**Sig.**
	**Regression coefficient B**	**SE**	**Beta**		
**(Constant)**	-46.539	41.940		-1.110	.268
**Defiance**	5.790	1.563	.229	3.704	**.000**
**Age**	-.457	.375	-.076	-1.218	.224
**Sex**	-6.217	9.353	-.041	-.665	.507
**Lung disease**	1.475	4.458	.020	.331	.741

F=3.72 (df=257), p=.006; adjusted R^2=.04, SE= standard error
